# Genomic surveillance of genes encoding the SARS-CoV-2 spike protein to monitor for emerging variants on Jeju Island, Republic of Korea

**DOI:** 10.3389/fmicb.2023.1170766

**Published:** 2023-07-18

**Authors:** Young-Ran Ha, Hyun-Jeong Kim, Jae-Sung Park, Yoon-Seok Chung

**Affiliations:** ^1^Jeju Branch Office, Honam Regional Center for Disease Control and Prevention, Korea Disease Control and Prevention Agency, Jeju, Republic of Korea; ^2^Jeju National Quarantine Station, Korea Disease Control and Prevention Agency, Jeju, Republic of Korea; ^3^Division of Infectious Disease Diagnosis Control, Honam Regional Center for Disease Control and Prevention, Korea Disease Control and Prevention Agency, Gwangju, Republic of Korea

**Keywords:** COVID-19, SARS-CoV-2, spike protein, Republic of Korea, Jeju Island

## Abstract

**Introduction:**

The severe acute respiratory syndrome coronavirus 2 (SARS-CoV-2) pandemic has been fueled by new variants emerging from circulating strains. Here, we report results from a genomic surveillance study of SARS-CoV-2 on Jeju Island, Republic of Korea, from February 2021 to September 2022.

**Methods:**

A total of 3,585 SARS-CoV-2 positive samples were analyzed by Sanger sequencing of the gene encoding the spike protein before performing phylogenetic analyses.

**Results:**

We found that the Alpha variant (B.1.1.7) was dominant in May 2021 before being replaced by the Delta variant (B.1.617.2) in July 2021, which was dominant until December 2021 before being replaced by the Omicron variant. Mutations in the spike protein, including N440K and G446S, have been proposed to contribute to immune evasion, accelerating the spread of Omicron variants.

**Discussion:**

Our results from Juju Island, Republic of Korea, are consistent with and contribute to global surveillance efforts crucial for identifying new variants of concern of SARS-CoV-2 and for monitoring the transmission dynamics and characteristics of known strains.

## Introduction

The coronavirus disease 2019 (COVID-19) is caused by numerous genetic variants of severe acute respiratory syndrome coronavirus 2 (SARS-CoV-2) that have emerged throughout the world (Kumar et al., 2021). The rapid genetic changes of SARS-CoV-2 have been influenced by various factors, including low levels of existing immunity to each new strain and the high mutation rates caused by error-prone polymerases (Denison et al., 2011). Screening for changes in genetic characterization could help predict vaccine effectiveness and vaccine-induced immune evasion to predict outbreaks (Mengist et al., 2021; Shaibu et al., 2021).

The SARS-CoV-2 genome encodes four structural proteins: nucleoprotein (NP), membrane (M), envelope (E), and spike (S) proteins (Rathnasinghe et al., 2021). SARS-CoV-2 invades host cells via direct contact with the human angiotensin-converting enzyme 2 (hACE2) receptor and the receptor binding domain (RBD) on the spike protein (Walls et al., 2020; Chen et al., 2022; Rashid and Salih, 2022). Spike (S) protein is a surface glycoprotein comprised of two functional subunits (i.e., S1 and S2). The S1 subunit is composed of an N-terminal domain and an RBD. The S2 subunit contains four domains: (1) a fusion peptide (FP) that mediates the binding of viral and host cell membranes; (2) two heptad repeat regions (HR1 and HR2) important for viral fusion; (3) a transmembrane (TM) domain that is role for membrane fusion activity; and (4) a cytoplasmic (CT) domain (Bosch et al., 2003; Corver et al., 2009; Guruprasad, 2021; Paul et al., 2021).

Upon binding to receptors on the cell surface, the spike protein is activated by TM protease serine 2 (TMPRSS2), promoting the entry of the virus into cells and making it an important target for multiple vaccines (Hoffmann et al., 2020). The protein has also been monitoring mutations, including insertions, deletions, and amino acid substitutions (Walls et al., 2020).

The World Health Organization (WHO) has been monitoring changes to the SARS-CoV-2 genome (World Health Organization [WHO], 2021), classifying emerging variants as variants of concern (VOC), variants of interest (VOI), and variants under monitoring (VUM) (World Health Organization [WHO], 2021). Since late November 2021, the Omicron (B.1.1.529) as a VOC has dominated global spread, replacing previously circulating VOCs, notably Alpha (B.1.1.7), Beta (B.1.531), and Gamma (P.1) by March 2022, and Delta (B.1.627.2) by July 2022 (World Health Organization [WHO], 2021). Since February 2022, the WHO began introduced a fourth variant category called “Omicron subvariants under monitoring,” which included the Omicron subvariants BA.5, BA.2.75, BA.4.6, XBB, and BA.2.3.20 by 29 November 2022 (World Health Organization [WHO], 2021). To be classified as an Omicron subvariants under monitoring, the strain must: (i) belong to a currently circulating VOC by phylogenetic analysis; (ii) be more transmissible than other circulating VOC lineages; and (iii) have amino acid substitutions that confer fitness advantages relative to other circulating variants, in epidemiology studies (World Health Organization [WHO], 2021).

In this study, we sequenced and analyzed 3,585 SARS-CoV-2 positive specimens isolated from COVID-19 positive patients on Jeju Island, Republic of Korea, from January 2021 to September 2022. We analyzed spike protein gene sequences and performed phylogenetic analyses that allowed us to follow the dynamics of Alpha, Beta, Delta, and Omicron variants in the region and report mutations related to known mechanisms of immune evasion.

## Materials and methods

### Epidemiological data analysis

**FIGURE 1:**
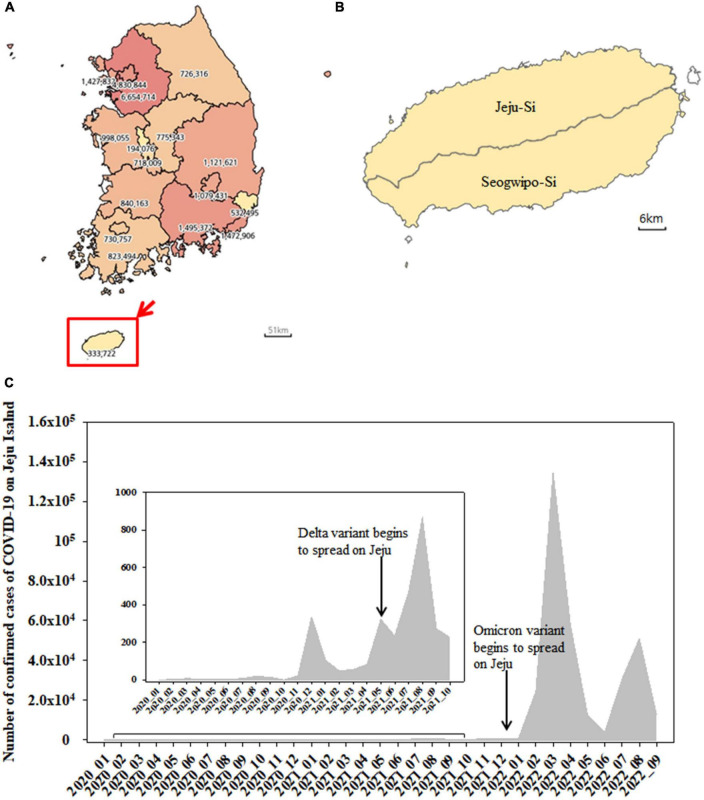
Map of South Korea showing the number of confirmed cases of COVID-19 from January 2020 to September 2022 **(A)**. Jeju Island including Jeju-Si and Seogwipo-Si **(B)**. The monthly number of confirmed cases of SARS-CoV-2 on Jeju Island from January 2020 to September 2022 **(C)**.

The map of South Korea was obtained from the Statistical Geographic Information Service (SIGS) offered by Statistics Korea^1^ (Figures 1A, B). The number of confirmed cases of COVID-19 was provided by Korea Disease Control and Prevention Agency (KDCA^2^) (Figure 1C).

### RNA isolation and reverse transcription-polymerase chain reaction

Coronavirus disease 2019 (COVID-19) positive specimens were obtained from Seoul Clinical Laboratories (SCL) and from the Jeju Special Self-Governing Province Institute of Environment Research. Specimens were handled in a Class II biosafety cabinet (Thermo Scientific 1300 Series A2) in a biosafety level 2 (BL2) laboratory. RNA was isolated using an RNA extraction kit (Qiagen, Valencia, CA, USA), as per the manufacturer’s instructions. cDNA was synthesized using SuperScript IV First-Strand Synthesis System (Invitrogen, Carlsbad, CA, USA), and PCR was conducted using PrimeSTAR GXL DNA Polymerase (Takara, Shiga, Japan) from January to August 2021. From September 2021 to September 2022, reverse transcription-polymerase chain reaction (RT-PCR) was conducted using Qiagen Onestep RT-PCR kit (Qiagen, Valencia, CA, USA), DiaStar 2 × OneStep RT-PCR premix kit (SolGent, Daejeon, Republic of Korea), and SEQMAX qPCR one step mastermix (Nine Korea, Republic of Korea). Sequences of primers used in RT-PCR are shown in Table 1. To detect mutations of SARS-CoV-2 spike protein, L71/R75, L76/R79, and L80/R84 primer sets were used. RT-PCR was performed under the following conditions: an initial reverse transcription step at 50°C for 30 min followed by a denaturation step at 95°C for 5–15 min. This was followed by 35 cycles of 30 s at 95°C, 30 s at 58°C, 1 min 30 s at 68°C, and a final extension step at 68°C for 7 min (Ha et al., 2022). To distinguish between BA.4 and BA.5, RT-PCR was instead performed on the N (nucleocapsid) gene under the following conditions: an initial reverse transcription step at 50°C for 30 min followed by a denaturation step at 95°C for 5–15 min. This was followed by 35 cycles of 10 s at 98°C, 15 s at 50°C, 2 min 30 s at 68°C, and a final extension step at 68°C for 7 min.

**TABLE 1:** List of primer sequences used for RT-PCR analyses of spike protein (Fragment 1, 2, and 3) and N protein (L94 and R95).

Fragment		Primer	Primer sequence	Binding position	Fragment size (bp)
S protein	Fragment 1	L71	Reverse: 5′-ACAAATCCAATTCAGTTGTCTTCCTATTC-3′	21,358–21,386	1,546
		L73	Reverse: 5′-CAATTTTGTAATGATCCATTTTTGGGTGT-3′	21,962–21,990	
		R73	Reverse: 5′-CACCAGCTGTCCAACCTGAAGA-3′	22,325–22,346	
		R75	Reverse: 5′-ACCACCAACCTTAGAATCAAGATTGT-3′	22,878–22,903	
	Fragment 2	L76	Forward: 5′-AGGGCAAACTGGAAAGATTGCT-3′	22,798–22,819	1,372
		L78	Forward: 5′-CAACTTACTCCTACTTGGCGTGT-3′	23,444–23,466	
		R77	Reverse: 5′-CAGCCCCTATTAAACAGCCTGC-3′	23,501–23,522	
		R79	Reverse: 5′-CATTTCATCTGTGAGCAAAGGTGG-3′	24,146–24,169	
	Fragment 3	L80	Forward: 5′-TTGCCTTGGTGATATTGCTGCT-3′	42,079–24,100	1,595
		L83	Forward: 5′-TCCTTTGCAACCTGAATTAGACTCA-3′	24,979–25,003	
		R82	Reverse: 5′-TGCCAGAGATGTCACCTAAATCAA-3′	25,053–25,076	
		R84	Reverse: 5′-AGGTGTGAGTAAACTGTTACAAACAAC-3′	25,647–25,673	
N gene		L94	Forward: 5′-GGCCCCAAGGTTTACCCAATAA-3′	28,395–28,416	669
		R95	Reverse: 5′-CAGTACGTTTTTGCCGAGGCTT-3′	29,042–29,063	

The position number of each primer sequence was compared to Wuhan Hu-1, genome sequence (accession number: NC_045512.2).

### DNA sequencing and analyses

DNA sequencing (Cosmogenetech, Seoul, Republic of Korea and SolGent, Daejeon, Republic of Korea) was performed using a standard protocol. To analyze the sequences for spike protein, L71/R75, L76/R79, and L80/R84 and inner primers including L73/R73, L78/R77, and L83/R82 were used until December 2021 (Table 1). From 2022, inner primers were used only L73, L78, and L83. To detect the mutation in the N gene, L94/R95 were used from July and September 2021. The sequence data from COVID-19 positive specimens on Jeju Island were submitted to GenBank through the National Center for Biotechnology Information (NCBI) (Table 2). Full-length sequences of COVID-19 spike proteins of various geographical origins were downloaded in FASTA format from GISAID. The sequence of SARS-CoV-2 reference genome Wuhan Hu-1 (accession number: NC 045512.2) was retrieved from the NCBI databases. The non-coding 3′ and 5′ regions were trimmed using CLC Genomic Workbench 5.0.1 software (CLC bio, Denmark). Multiple sequence alignments were done using a multiple sequence alignment (MAFFT) programs. Sequences of SARS-CoV-2 with specific mutations were searched in the GISAID database.^3^ Phylogenetic analysis was done using iTOL.^4^ Branch support was calculated by bootstrap, consisting of 1,000 alignments. SARS-CoV-2 positive specimens from confirmed cases of Jeju Island were classified into clades and lineages using Nextclade Beta.^5^

**TABLE 2:** Spike protein mutations from SARS-CoV-2 confirmed cases of Jeju Island including Alpha, Beta, Delta, and Omicron.

Variant	GenBank accession no.	Lineage/ Nextclade	Length (nt)	Existing mutation in spike protein	Deletions
Alpha variant	OP763726∼ OP763743	B.1.1.7/20I	3,822	A570D, S982A, P681H, D614G, S98F, T716I, Y144del, N501Y, D138H, D1118H, V70del, H69del	21,765–21,770, 21,992–21,994
Beta variant	OP763744	B.1.351/20H	3,822	D614G, D215G, D80A, L244del, A67V, L18F, N501Y, A701V, E484K, L242del, A243del	22,283–22,291
	OP763745	B.1.351/20H	3,822	D614G, D215G, D80A, H49Y, L244del, K417N, A27S, N501Y, A701V, E484K, L242del, A243del	22,283–22,291
Delta variant	OP763699∼ OP763702	B.1.617.2/21I	3,822	T19R, P681R, D614G, D950N, T478K, L452R, F157del, R158del, E156G, A222V, G142D, A475S	22,029–22,034
	OP763704	B.1.617.2/21I	3,835	T19R, P681R, D614G, D950N, T478K, L452R, F157del, R158del, E156G, A222V, G142D, V289L	22,029–22,034
	OP763703, OP763705∼ OP763707	B.1.617.2/21I	3,822	T19R, P681R, D614G, D950N, T478K, L452R, F157del, R158del, E156G, A222V, G142D	22,029–22,034
	OP763708	B.1.617.2/21A	3,822	T19R, P681R, D614G, D950N, T478K, L452R, F157del, R158del, E156G, A222V, G142D, T95I	22,029–22,034
	OP763709	B.1.617.2/21A	3,822	T19R, P681R, D614G, D950N, T478K, L452R, F157del, R158del, E156G, A222V, G142D, A475S	22,029–22,034
	OP763711	B.1.617.2/21A	3,824	T19R, P681R, D614G, D950N, T478K, L452R, F157del, R158del, E156G, G142D, S255F	22,029–22,034
	OP763712∼ OP763714	B.1.617.2/21A	3,816	T19R, P681R, D614G, D950N, T478K, L452R, F157del, R158del, E156G, G142D	22,029–22,034
	OP763715	B.1.617.2/21A	3,824	T19R, P681R, D614G, D950N, T478K, L452R, F157del, R158del, E156G, G145D	22,029–22,034
	OP763716∼ OP763719	B.1.617.2/21A	3,824	T19R, P681R, D614G, D950N, T478K, L452R, F157del, R158del, E156G, G143D, S255F	22,029–22,034
Omicron variant	OP763667∼ OP763668	BA.1.1/21K	3,833	N679K, Q493R, Y145del, G339D, G446S, P681H, D614G, N969K, R346K, N764K, T478K, H655Y, G496S, N856K, N440K, Y144del, N211del, ins214EPE, A67V, S371L, Q498R, T547K, L981F, S375F, Q954H, S477N, N501Y, T95I, G142D, Y505H, D796Y, V143del, V70del, S373P, L212I, E484A, H69del	21,765–21,770, 21,987–21,995, 22,194–22,196
	OP763669	BA.1.1/21K	3,834	N679K, Q493R, Y145del, G339D, G446S, P681H, D614G, N969K, R346K, N764K, T478K, H655Y, G496S, N856K, N440K, Y144del, N211del, ins214EPE, A67V, S371L, Q498R, L981F, S375F, Q954H, S477N, N501Y, T95I, G142D, Y505H, D796Y, V143del, V70del, S373P, L212I, E484A, H69del	21,765–21,770, 21,987–21,995, 22,194–22,196
	OP763670∼ OP763671	BA.1.1/21K	3,833	N679K, Q493R, Y145del, G339D, G446S, P681H, D614G, N969K, R346K, N764K, T478K, H655Y, G496S, N856K, N440K, Y144del, N211del, ins214EPE, A67V, S371L, Q498R, K417N, T547K, L981F, S375F, Q954H, S477N, N501Y, T95I, G142D, Y505H, D796Y, V143del, V70del, S373P, L212I, E484A, H69del	21,765–21,770, 21,987–21,995, 22,194–22,196
	OP763672, OP763674∼ OP763675	BA.2/21L	3,833	V213G, N679K, T376A, Q493R, D405N, G339D, R408S, P681H, D614G, N969K, N764K, T478K, H655Y, N440K, Q498R, K417N, S371F, S375F, Q954H, S477N, N501Y, G142D, Y505H, D796Y, S373P, E484A	–
	OP763673, OP763676∼ OP763679	BA.2/21L	3,838	V213G, N679K, T376A, Q493R, D405N, G339D, R408S, P681H, D614G, N969K, T478K, H655Y, N440K, Q498R, K417N, S371F, S375F, Q954H, S477N, N501Y, G142D, Y505H, D796Y, S373P, E484A	–
	OP763680	BA.2/21L	3,837	V213G, N679K, T376A, Q493R, D405N, G339D, R408S, P681H, D614G, N969K, T478K, H655Y, N440K, Q498R, S371F, S375F, Q954H, S477N, N501Y, G142D, Y505H, D796Y, S373P, E484A	–
	OP763682	BA.2.12.1/22C	3,770	V213G, S704L, N679K, T376A, Q493R, D405N, G339D, R408S, P681H, D614G, N969K, N764K, T478K, H655Y, N440K, Q498R, K417N, S371F, S375F, Q954H, S477N, N501Y, G142D, Y505H, D796Y, L452Q, S373P, A879V, E484A	–
	OP763683	BA.2.12.1/22C	3,749	V213G, S704L, N679K, T376A, Q493R, D405N, G339D, R408S, P681H, D614G, N969K, N764K, T478K, H655Y, Q498R, S371F, S375F, Q954H, S477N, N501Y, G142D, Y505H, D796Y, L452Q, S373P, E484A	–
	OP763685	BA.2.12.1/22C	3,770	V213G, S704L, N679K, T376A, Q493R, D405N, G339D, R408S, P681H, D614G, N969K, N764K, T478K, H655Y, N440K, Q498R, S371F, S375F, Q954H, S477N, N501Y, Y505H, D796Y, L452Q, S373P, A879V, E484A	–
	OP763686	BA.2.12.1/22C	3,750	V213G, S704L, N679K, T376A, Q493R, D405N, G339D, R408S, P681H, D614G, N969K, N764K, T478K, H655Y, N440K, Q498R, S371F, S375F, Q954H, S477N, N501Y, G142D, Y505H, D796Y, L452Q, S373P, E484A	–
	OP763688	BA.5/21L	3,741	V213G, N679K, T376A, D405N, G339D, F486V, L179P, R408S, P681H, D614G, N969K, N764K, T478K, L452R, H655Y, N440K, Q498R, K417N, S371F, S375F, Q954H, S477N, N501Y, G142D, Y505H, D796Y, V70del, S373P, E484A, H69del	21,765–21,770
	OP763687, OP763689∼ OP763694	BA.5/21L	3,741	V213G, N679K, T376A, D405N, G339D, F486V, R408S, P681H, D614G, N969K, N764K, T478K, L452R, H655Y, N440K, Q498R, K417N, S371F, S375F, Q954H, S477N, N501Y, G142D, Y505H, D796Y, V70del, S373P, E484A, H71del	21,765–21,770
	OP763695	BA.5/21L	3,741	V213G, N679K, T376A, D405N, G339D, F486V, R408S, P681H, D614G, N969K, N764K, T478K, L452R, H655Y, N440K, Q498R, S371F, S375F, Q954H, S477N, N501Y, G142D, Y505H, D796Y, V70del, Q271R, S373P, E484A, H69del	21,765–21,770
	OP763696∼ OP763697	BA.1.1/21K	3,834	N679K, Q493R, Y145del, G339D, G446S, P681H, D614G, N969K, R346K, N764K, T478K, H655Y, G496S, N856K, N440K, Y144del, N211del, ins214EPE, A67V, S371L, Q498R, K417N, T547K, L981F, S375F, Q954H, S477N, N501Y, T95I, G142D, Y505H, D796Y, V143del, V70del, S373P, L212I, E484A, H69del	21,765–21,770, 21,987–21,995, 22,194–22,196
	OP763698	BA.1.1/21K	3,833	N679K, Q493R, Y145del, G339D, G446S, P681H, D614G, N969K, R346K, N764K, T478K, H655Y, G496S, N856K, N440K, Y144del, N211del, ins214EPE, A67V, S371L, Q498R, T547K, L981F, S375F, Q954H, S477N, N501Y, T95I, G142D, Y505H, D796Y, V143del, V70del, S373P, L212I, E484A, H69del	21,765–21,770, 21,987–21,995, 22,194–22,196

Additional mutations are marked in red.

## Results

### Epidemiological data analysis

Jeju Island is Korean Republic’s biggest Island, located 80 km away from the mainland (Jang et al., 2019) and includes Jeju-Si and Seogwipo-Si (Figures 1A, B). The number of confirmed cases of COVID-19 was shown from January 2020 to September 2022 (Figure 1A). The cumulative number of COVID-19 cases in Jeju was 333,722, accounting for 1.35% of the total number of cases in the Republic of Korea (Figure 1A). From January 2020 to September 2022, the monthly number of confirmed cases of SARS-CoV-2 on Jeju Island was shown in Figure 1C. The Delta (B.1.627.2) variant emerged and began to spread on the island in May 2021, with a sharp increase in confirmed cases appearing later (e.g., from July to August 2021) (Figure 1C). The Omicron (B.1.1.529) variant was first detected in December 2021 and confirmed cases rose beginning shortly after (e.g., January 2022) (Figure 1C).

### Distribution of clades

**FIGURE 2:**
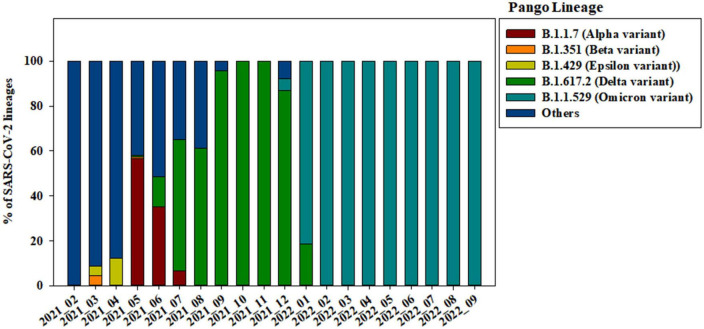
Changes in the genomic prevalence of SARS-CoV-2 variants characterized by spike protein mutations from February 2021 to September 2022 across Jeju Island.

Of the 3,585 spike protein gene sequences analyzed in our study, 3,322 (92.66%) were local and 263 (7.34%) were imported from off-island (Supplementary Figure 1). SARS-CoV-2 variants were classified into lineages based on the sequences of their spike protein genes (Figure 2). From April to September 2021, strains from clade 20A (i.e., B.1.619, B.1.619.1, and B.1.620) were also observed. B.1.619 lineage was first observed in April 2021 and 20.00, 32.43, and 31.52% from May to July 2021. B.1.620 lineage also observed in April 2021 and 4.83 and 18.92% in May and June 2021 (Supplementary Figure 2). The prevalence of Epsilon (B.1.429), one of the previous circulating VOIs, was 4.35 and 12.24% in March and April 2021, respectively (Figure 2).

Alpha variant (B.1.1.7) has several amino acid exchanges and deletions within the viral spike protein including del69–70HV, del144Y, N501Y, A570D, D614G, P681H, T761I, S982A, and D1118H (Socher et al., 2021). In this study, Alpha variant (OP763726∼OP763743) has an additional spike S98F and D138H mutations, which are present in 1.90 and 1.14% of strains deposited in the GISAID database, as of May 2023 (Table 2). Previous study also noted that S98F, D138H mutation was found in in less than 2% of genomes worldwide (Zarate et al., 2022). The S98F and D138H mutations are also found in Alpha variant such as hCoV-19/Spain/AN-CBA-06831/2021 (EPI_ISL_15678545), hCoV-19/Germany/BW-RKI-I-1053239/2021 (EPI_ISL_16147015), hCoV-19/USA/PA-CDC-4197450/2021 (EPI_ISL_17534404), and hCoV-19/Chile/AP-ISPC-86703/2021 (EPI_ISL_17028381).

The previously circulating VOCs, Beta variant (B.1.351) was detected infrequently, its prevalence decreasing from 4.35 to 0.69% between March and May 2021 (Figure 2). Beta variant contains amino acid mutations within the viral spike protein including L18F, D80A, D215G, deletion at positions 242–244 (L242del, A243del, and L244del), K417N, E484K, N501Y, A701V, A27S, and D614G (Edara et al., 2021; Sanches et al., 2021; Velasco et al., 2021). In this study, Beta variant (OP763744∼OP763745) has an additional spike A67V and H49Y mutations, which are present in 1.35 and 0.11% of strains deposited in the GISAID database, as of May 2023 (Table 2). The A67V mutations are also found in Beta variant such as hCoV-19/Canada/UN-63124/2021 (EPI_ISL_12388094), hCoV-19/Bangladesh/icddrb-GAC2-285/2021 (EPI_ISL_13477157), hCoV-19/USA/IN-CDC-LC26926/2021 (EPI_ISL_17532096), and hCoV-19/Spain/GA-CHUVI-19298619c-2/2021 (EPI_ISL_176218 27). The H49Y mutations are also found in Beta variant such as hCoV-19/South_Africa/NICD-K253-002-172/2021 (EPI_ISL_1607 8823), hCoV-19/South_Korea/KDCA2733s/2021 (EPI_ISL_27120 10), hCoV-19/USA/NY-CDC-QDX24758501/2021 (EPI_ISL_436 7140), and hCoV-19/Philippines/PH-PGC-43487/2021 (EPI_ ISL_5569124).

The Delta variant was first observed in May 2021 and began to dominate from July to December 2021, while Omicron emerged and replaced previous strains starting in January 2022 (Figure 2). The spike gene mutations in Delta variant are T19R, L452R, T478K, D614G, P681R, E156G, G142D, and D950N, with deletions at positions 157 and 158 (Shiehzadegan et al., 2021; Dhawan et al., 2022; Focosi et al., 2022; Singh et al., 2022b). In this study, Delta variant (OP763715) has an additional spike G145D mutation, which are present in 0.01% of strains deposited in the GISAID database, as of May 2023 (Table 2). The G145D mutations was also found in Delta variant such as hCoV-19/USA/CA-HLX-STM-3XXQ3BM69/2021 (EPI_ISL_12833436), hCoV-19/India/TN-INSACOG-CBR-S-7676/2021 (EPI_ISL_14677988), and hCoV-19/Russia/MOW-PMVL-DZ-19421141784/2021 (EPI_ ISL_15884308).

**FIGURE 3:**
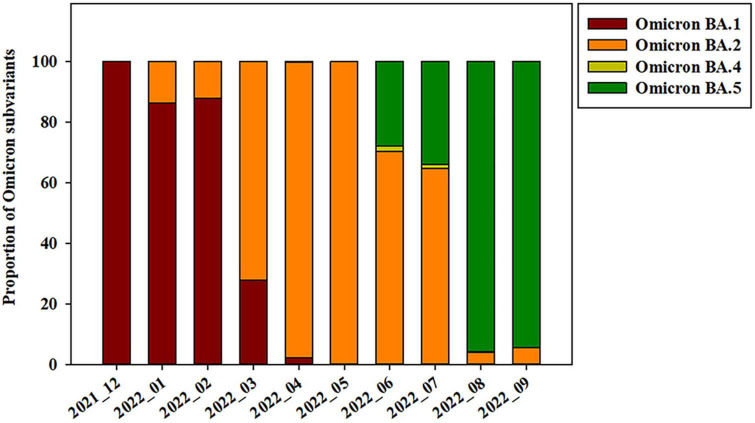
Prevalence of SARS-CoV-2 Omicron subvariants on Jeju Island from December 2021 to September 2022.

The Omicron variant was first observed in the case of overseas imports in December 2021. It became dominant in 2022, before being supplanted by the BA.2 subvariant between January and April 2022 (Figures 2, 3). BA.4 and BA.5 begin to replace the BA.2 variant beginning in June 2022, while the BA.5 subvariant dominated transmission from August to September 2022 (Figure 3). Between August and September 2021, the prevalence of BA.5 increased from 27.84 to 34.05%. The spike gene mutations in Omicron variant are A67V, Δ69-70, T95I, G142D/Δ143-145, Δ211/L212I, ins214EPE, V213G, L212I, G339D, S371L, S371F, S373P, S375F, T376A, D405N, R408S, K417N, N440K, G446S, S477N, T478K, L452R/L452Q, E484A, F486V, Q493R, G496S, Q498R, N501Y, Y505H, T547K, D614G, H655Y, N679K, P681H, S704L, N764K, D796Y, N856K, A879V, Q954H, N969K, and L981F (Muik et al., 2022; Shanmugaraj et al., 2022; Singh et al., 2022a; Takashita et al., 2022; Yoshida et al., 2022). In this study, Omicron variant (OP763688 and OP763695) has an additional spike L179P and Q271R mutations, which are present in 0.02 and 0.002% of strains deposited in the GISAID database, as of May 2023 (Table 2). The Q271R mutation was also found in Omicron variant such as hCoV-19/Sweden/C-03_SE600_000200436976N2/2022 (EPI_ISL_17712759), hCoV-19/Canada/QC-L00612610001/2023 (EPI_ISL_17641597), and hCoV-19/South_Korea/GJ-HERI-A1144/2022 (EPI_ISL_17703223). The Q271R mutation was also found in Omicron variant such as hCoV-19/France/ARA-HCL-723000653001/2023 (EPI_ISL_17657463), hCoV-19/Brazil/PA-LACENPA-150377499/2023 (EPI_ISL_17514895), and hCoV-19/South_Korea/KDCA227335/2023 (EPI_ISL_17473659).

### Phylogenetic analyses of currently and previously circulating VOCs

**FIGURE 4:**
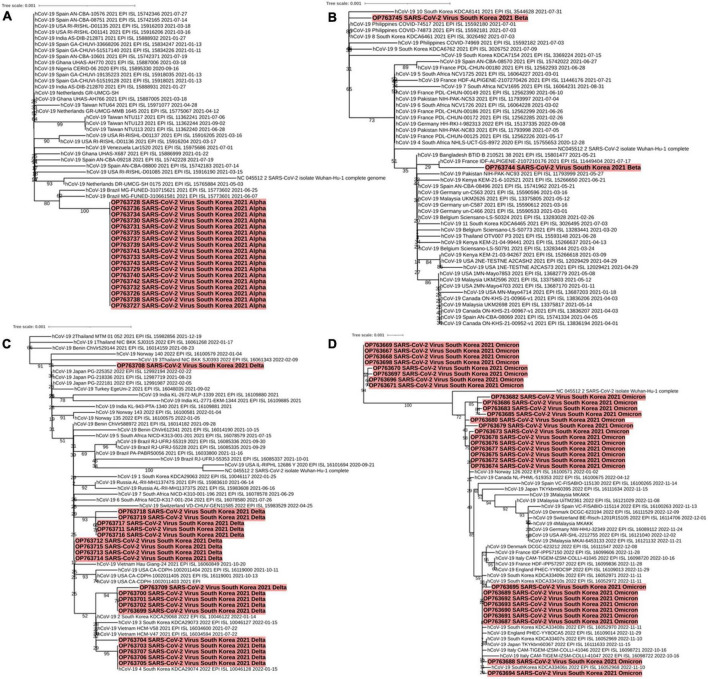
Phylogenetic analysis of SARS-CoV-2 variants including Alpha **(A)**, Beta **(B)**, Delta **(C)**, and Omicron **(D)** characterized by spike protein mutations from December 2021 to September 2022 across Jeju Island. Red parts indicate the genomes from confirm cases in Jeju Island.

A phylogenetic tree of Alpha (B.1.1.7) variants isolated from patients on Jeju Island between May and early June 2021 (OP763726∼OP763643) was generated. The Alpha lineage from samples isolated on Jeju Island was more closely related to each other than the rest of Alpha lineage (Figure 4A). Similarly, we generated phylogenetic trees of samples isolated from patients on Jeju Island for Beta (B.1.531, March to May 2021, OP763744 and OP763645). Notably, the sequence from sample OP763744 closely resembled that of hCoV-19/France/IDF-ALPIGENE-2107210176 2021 (EPI ISL 11449404) (Figure 4B). The Delta variant on Jeju Island was grouped by confirmation date of COVID-19 infection (Figure 4C). The sequences from OP763699 to OP763709 emerged August and September 2021, and Nextclade analyses classified these as belonging to clade 20I. Notably, the sequences from OP763703 to OP763707 closely match that of hCoV-19/South Korea/KDCA29074/2022 (EPI ISL 10046128). The sequences from OP763711 to OP763719 emerged in November 2021, and Nextclade analyses classified these to clade 21A. We also report that in OP763708, an additional T95I mutation was found in Spike protein (Table 2). The genomes sequences of B.1.1.529 (Omicron) on Jeju Island clustered several subvariants including BA.1.1, BA.2, and BA.5 (Figure 4D).

The sequences from OP763667 to OP763671, OP763696, and OP763698 were emerged in March 2022. These sequences belong to BA.1.1 subvariant. The sequences from OP763672 to OP763681 were also emerged on March 2022 and belong to BA.2 subvariant. The sequences from OP763682, OP763683, OP763685, and OP763686 were emerged on March 2022 and belong to BA.2.12.1. The sequences from OP763687 to OP753695 were emerged in August 2022 and belong BA.5 subvariant (Figure 4D). The sequence from OP763672 to OP763680, OP763682, OP763683, OP763685, and OP763686 closed to hCoV-19/Norway 126 2022 (EPI ISL 16100571) and hCoV-19/Canada NL-PHML-519353 2022 (EPI ISL 16100675). The sequence of OP753695 closed to hCoV-19/South Korea/KDCA33410s/2022 (EPI ISL 16052972). The number of mutations in Spike protein of Alpha, Beta, and Delta variant ranged from 11 to 12, while the number of mutations of Omicron exceeded 30 (Table 2).

## Discussion

In this study, we performed genomic surveillance of SARS-CoV-2 samples isolated from patients on Jeju Island 1 year after that original infection, from February 2021 to September 2022, a period during which multiple variants were circulating globally. During the period of our spike protein-based genomic surveillance study on Jeju Island, the Alpha, Delta, and Omicron variants (i.e., currently and previously circulating VOCs) became dominant in turn, while the Beta and Gamma variants did not. In addition, the import and prevalence of mutations in Jeju showed different from the mainland of Korea.

On Jeju Island, the Alpha variant was still dominant in May 2021 before decreasing from 56.55 to 6.52% as the Delta variant increased in circulation (Figure 2). In the Republic of Korea, Alpha variant was decreased from 27.40 to 8.1% in May and July 2021 (Kim et al., 2021). Globally, the Alpha variant dominated transmission until the emergence of the Delta variant in the winter of 2020 (Dong et al., 2022). Our data suggests that the Delta variant began to spread on overseas imported case from Jeju on May 2021, with confirmed cases of COVID-19 increasing that month from 82 to 323. After that, confirmed cases of COVID-19 on Jeju Island increased from 468 to 870 from July to August 2021 (Figure 1C and Supplementary Figure 3). In the Republic of Korea, the Delta variant was first confirmed on April 2021 and exceeded half of all cases by July 2021 (Kim et al., 2021, 2022). Globally, the Delta variant has been replaced by the Omicron variant by the end of 2021 (Lista et al., 2022), while on Jeju Island, Delta variant has been dominant over half the year, only being replaced by Omicron in January 2022 (Figure 2).

The Omicron variant was declared as a VOC on 26 November 2021, after being identified earlier that month in South Africa (Gobeil et al., 2022). In the Republic of Korea, the Omicron variant was first confirmed in the end of November 2021 and exceeded half of all cases on December 2021 (Kim et al., 2022). By February 2022, the Omicron variant accounted for 99.1% of all COVID-19 cases in the Republic of Korea (Park et al., 2023). On Jeju Island, confirmed cases of COVID-19 increased significantly in a short period, from 784 in January 2022 to 134,559 in April 2022 (Figure 1C). In addition, the Omicron variant accounted for more than 99.00% of all COVID-19 cases on February 2022 (Figure 2). From this result, the import and prevalence of mutations in Jeju are different from those in Korea.

## Conclusion

In conclusion, we used genomic surveillance and phylogenetic analyses to follow the relative abundance of different SARS-CoV-2 variants, from January 2020 to February 2022, on Jeju Island, Republic of Korea. During this period, multiple variants and their subvariants emerged having enhanced human-to-human transmissibility. The dynamics of variants differed on Jeju Island compared with the mainland. Because of the limited entry points compared to land-locked regions, new variants might be introduced more slowly or take different forms due to other Island characteristics. Being a major center for domestic and international travel, Jeju Island might be at risk of continuous influx of new variants. We conclude that monitoring efforts for COVID-19 cases and SARS-CoV-2 variants is essential for monitoring and controlling outbreaks on Jeju Island.

## Data availability statement

The datasets presented in this study can be found in the GenBank. The accession number from OP763667 to OP763745 can be found in the article/Supplementary material.

## Ethics statement

This study uses strains obtained from Jeju Special Self-Governing Province Research Institute of Public Health and Environment and Seoul Clinical Laboratories (SCL). Korea Disease Control and Prevention Agency (KDCA) did not require the study to be reviewed or approved by an ethics committee because this study belongs to the case including (1) insignificant impact on donors of human derivatives and the public, (2) research in which researchers do not collect or record personal information, and (3) cases where human material is not directly collected. The Institutional Review Board (IRB) of Korea Disease Control and Prevention Agency (KDCA) granted exemption for this study (IRB exemption number: KDCA-2023-06-01-PE-01).

## Author contributions

Y-RH contributed to the conception, design, data acquisition, and drafting of the manuscript. H-JK conducted the experiment and helped sequencing data analysis. J-SP advised the experimental procedure and treatment of specimens. Y-SC conceived the entire study and helped draft the manuscript. All authors read and approved the final manuscript.

## Footnotes

1
https://sgis.kostat.go.kr/

2
http://ncov.kdca.go.kr

3
https://www.gisaid.org/

4
https://itol.embl.de

5
http://clades.nextstrain.org/
