# Supplementary material for: Genomic surveillance of genes encoding the SARS-CoV-2 spike protein to monitor for emerging variants on Jeju Island, Republic of Korea
Source: Front Microbiol. 2023 Jul 18;14:1170766. doi: 10.3389/fmicb.2023.1170766 (PMC10390832; doi:10.3389/fmicb.2023.1170766)
Supplement: Supplementary file 1 [file Data_Sheet_1.pdf]

frontiers

# Supplementary Material

### *Epidemiological data analysis in domestic and imported cases*

The number of confirmed cases of COVID-19 was provided by Korea Agency of Disease Control and Prevention (KDCA, http://ncov.kdca.go.kr). A total of 3,585 SARS-CoV-2 positive samples were analyzed by Sanger sequencing of the gene encoding the spike protein from February 2021 to September 2022. In this study, 3,585 spike protein gene sequences analyzed, 3,322 (92.66%) were local and 263 (7.34%) were imported from off-island (Supplementary Figure 1). B.1.619 lineage was observed in April 2021 and 20.00%, 32.43% and 31.52% from May to July 2021. B.1.620 lineage observed in April 2021 and 4.83% and 18.92% in May and June 2021. Beta variant (B.1.351) was detected infrequently, its prevalence decreasing from 4.35% to 0.69% between March and May 2021 (Supplementary Figure 2). The Delta variant (B.1.617.2) began to spread on Jeju Island in overseas imported case in May 2021. In imported cases, Delta variant become dominant from May to November 2021, while Omicron (B.1.1.529) emerged and replaced previous strains starting in January 2022 (Supplementary Figure 3).

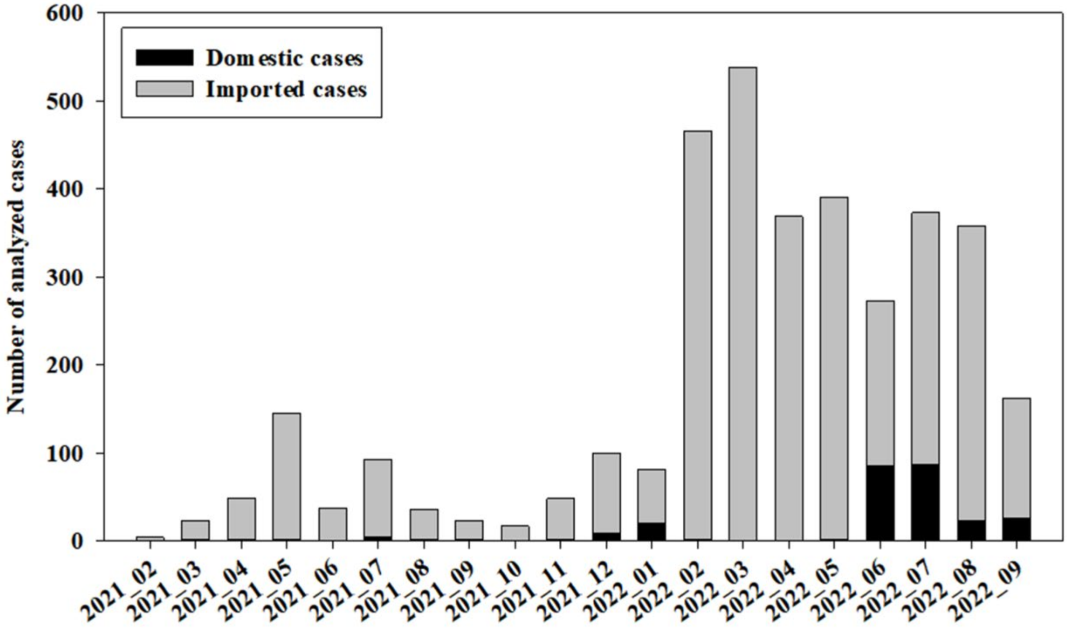


**Supplementary Figure 1.** Number of analyzed cases from February 2021 to September 2022.

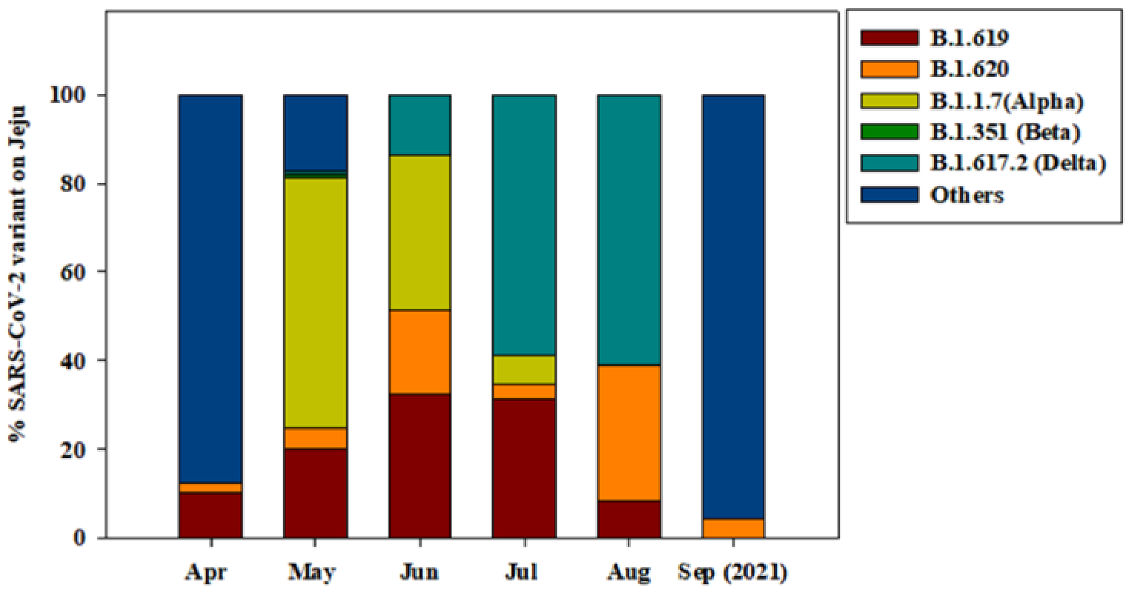


**Supplementary Figure 2.** Prevalence of SARS-CoV-2 variants characterized by spike protein mutations from April 2021 to September 2021 across Jeju Island

.

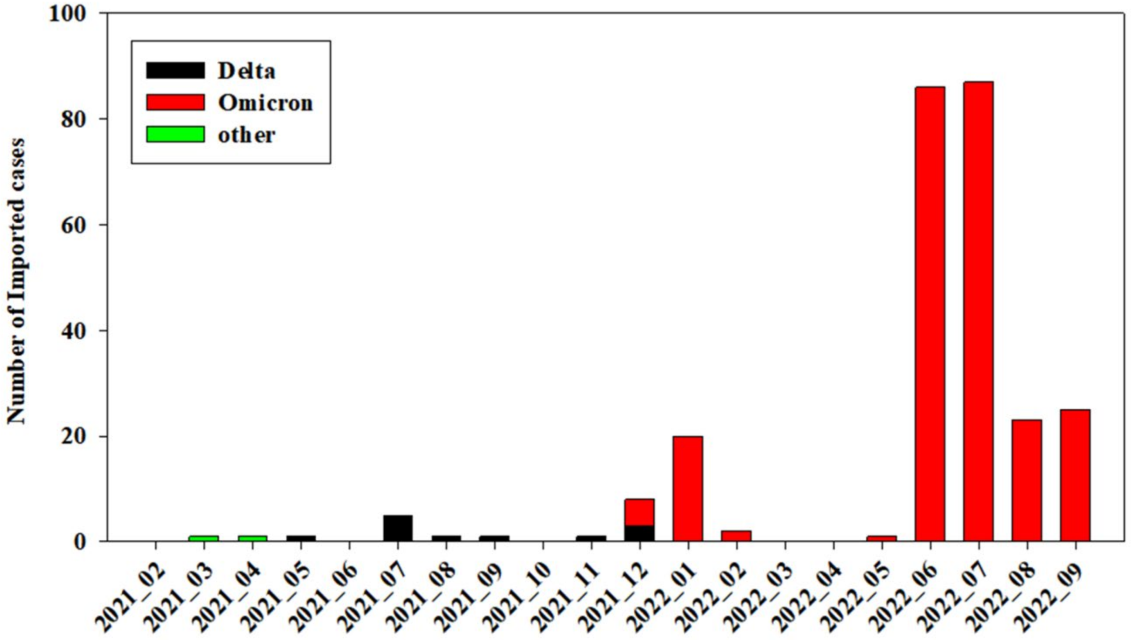


**Supplementary Figure 3.** Number of imported cases from February 2021 to September 2022 on Jeju Island among analyzed cases

.
